# LKB1 acts as a critical gatekeeper of ovarian primordial follicle pool

**DOI:** 10.18632/oncotarget.6792

**Published:** 2015-12-29

**Authors:** Zong-Zhe Jiang, Meng-Wen Hu, Xue-Shan Ma, Heide Schatten, Heng-Yu Fan, Zhen-Bo Wang, Qing-Yuan Sun

**Affiliations:** ^1^ State Key Laboratory of Reproductive Biology, Institute of Zoology, Chinese Academy of Sciences, Beijing, China; ^2^ University of Chinese Academy of Sciences, Beijing, China; ^3^ Department of Veterinary Pathobiology, University of Missouri, Columbia, Missouri, USA; ^4^ Life Science Institute, Zhejiang University, Hangzhou, China

**Keywords:** liver kinase B1 (LKB1), AMP-activated kinase (AMPK), mTOR complex (mTORC), ovary, POF

## Abstract

Liver Kinase b1 (LKB1/STK11)is a tumor suppressor responsible for the Peutz-Jeghers syndrome, an autosomal-dominant, cancer-prone disorder in which patients develop neoplasms in several organs, including the oviduct, ovary, and cervix. Besides, the C allele of a SNP in the *Lkb1* gene impedes the likelihood of ovulation in polycystic ovary syndrome (PCOS) in women treated with metformin, a known LKB1-AMPK activator. It is very likely that LKB1 plays roles in female fertility. To identify the physiological functions of LKB1 in the mouse ovary, we selectively disrupted LKB1 in oocytes by the *Cre-LoxP* conditional knockout system and found that *Lkb1^fl/fl^*; *Gdf9-Cre* mice were severely subfertile with significantly enlarged ovaries compared to *Lkb1^fl/fl^* mice. Interestingly, without *Lkb1* expression in oocytes from the primordial follicle stage, the entire primordial follicle pool was activated but failed to mature and ovulate, subsequently causing premature ovarian failure (POF). Further investigation demonstrated that elevated mTOR signaling regulated by an AKT-independent LKB1-AMPK pathway was responsible for the excessive follicle activation and growth. Our findings reveal the role of LKB1 as an indispensable gatekeeper for the primordial follicle pool, offer new functional understanding for the tumor suppressor genes in reproductive organs, and might also provide valuable information for understanding POF and infertility.

## INTRODUCTION

In the mammalian ovary, the majority of primordial follicles is maintained in dormancy and serve as the source of ova for the entire reproductive life [1, 2]. To ensure the proper reproductive lifespan, only a limited number of primordial follicles are recruited into the growing follicle pool through a process termed initial recruitment or follicular activation, which is a progressive and precisely regulated process [3]. Once activated, primordial follicles undergo a series of follicular development; only a few reach the preovulatory stage and release fertilizable eggs. Menopause occurs when the primordial follicle pool is exhausted [4]. Still, the molecular mechanisms underlying follicular recruitment are poorly defined. Recently, studies using genetically modified mouse models have revealed that the PTEN/PI3K/AKT signaling pathway is important for the regulation of follicular activation and survival [5]. The present study demonstrates an unexpected role for LKB1 in follicular activation.

LKB1 is a multifunctional serine/threonine kinase, involved in the regulation of cell energy homeostasis, establishment of cell polarity, vascular development, and tumor suppression [6]. LKB1 regulates energy homeostasis through the AMP-activated protein kinase (AMPK), whose threonine-172 on the catalytic α-subunit could be phosphorylated by LKB1 [7]. In humans, germ line mutations in *LKB1* are associated with Peutz-Jeghers syndrome (PJS) characterized by a predisposition to gastrointestinal neoplasms marked by a high risk of developing cancerous lesions in various organs [8]. Conditional knockout of *Lkb1* in pancreatic, vascular, neural and skeleton tissue revealed that LKB1 performs tissue specific actions in many organ systems [9-11].

In this study, we aimed to determine the physiological functions of LKB1 in the ovary by its conditional ablation from the oocytes in primordial and further developed follicles. We found that LKB1 was critical for female fertility and oocyte-specific deletion of Lkb1 induced overactivation of the primordial follicle pool from the time of puberty, resulting in follicular depletion and premature ovarian failure (POF) in adulthood. Strikingly, rapamycin blocked the overactivation of primordial follicles in Lkb1CKO ovaries. Furthermore, the elevated mTORC1 signaling was responsible for disrupted follicular activation in mutant mice. In addition, overactivated follicles could not develop to mature follicles and failed to ovulate because of defective follicular selection.

## RESULTS

### Disruption of *Lkb1* in oocytes from the primordial follicle stage causes premature follicular activation

**Figure 1:**
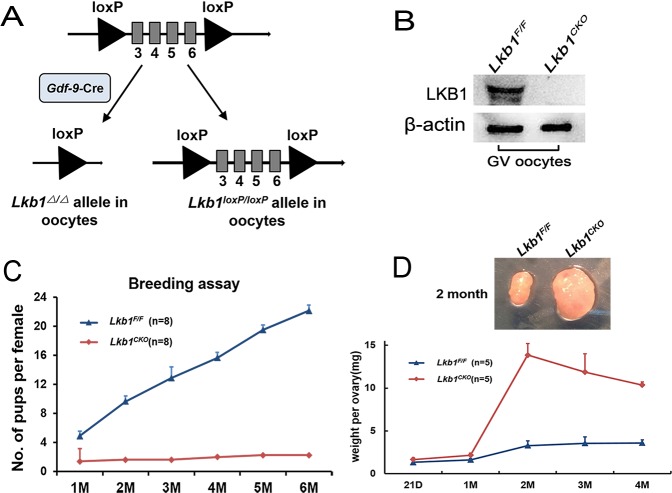
Lkb1 is essential for female fertility **A**. Schematic representation of deletion of Lkb1 exons and creation of Lkb1 Δ allele by Gdf-9-Cre-mediated recombination in oocytes. **B**. Western blots showing the knockout of LKB1 from mouse oocytes in *Lkb1^fl/fl^;Gdf9-Cre*. Oocytes were isolated from ovaries of 2-month-old mice and used for western blot. For each experiment, a sample from 10 *Lkb1^fl/fl^* or 3 *Lkb1^fl/fl^;Gdf9-Cre* mice was used. For each lane, 300 oocytes were used. **C**. Breeding assay shows subfertility of the female *Lkb1^fl/fl^;Gdf9-Cre* mice. Comparison of the cumulative number of progeny per female *Lkb1^fl/fl^* and *Lkb1^fl/fl^;Gdf9-Cre* mouse is shown. **D**. Ovary weights of *Lkb1^fl/fl^* and *Lkb1^fl/fl^;Gdf9-Cre* females at different ages and ovary sizes at 2 months. Data are shown as mean ± SEM.

The mutant mice, in which exons 3-6 of the *Lkb1* gene are targeted [12], were generated by crossing *Lkb1^fl/fl^* mice with transgenic mice expressing *Gdf9* promoter-mediated Cre recombinase [5] (Figure 1A). In *Gdf9-Cre* mice, Cre is expressed in oocytes of primordial follicles after postnatal day 3 and in later developmental stages [13]. Successful depletion of LKB1 protein in mouse oocytes was confirmed by western blot analysis (Figure 1B).

**Figure 2:**
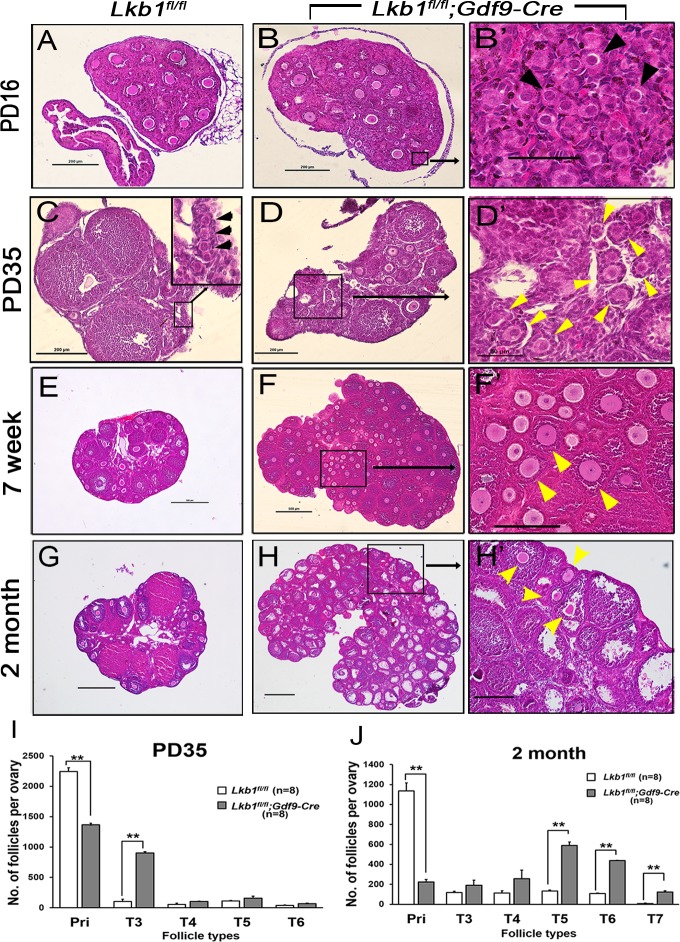
Premature activation of primordial follicle pool in *Lkb1^fl/fl^;Gdf9-Cre* mice **A**.-**H**. Histology of ovarian sections from *Lkb1^fl/fl^* and *Lkb1^fl/fl^;Gdf9-Cre* females of PD16, PD35, 7-week and 2-month old, respectively, stained with hematoxylin and eosin. Panels B', D', F' and H' are magnified images of rectangular areas marked with a solid line in panels **B**., **D**., **F**. and **H**., respectively. Black arrowheads in B' and C point to primordial follicles; yellow arrowheads in D', F' and H' show activated follicles. Bars: 50μm in B' and D'; 200μm in **A**.-**D**., F' and H'; 500μm in **E**.-**H**. **I**.-**J**. Shown are the quantifications of numbers of different types of follicles per ovary at the age of PD35 and 2 months, respectively. Ovaries from *Lkb1^fl/fl^* and *Lkb1^fl/fl^; Gdf9-Cre* mice with indicated ages were embedded in paraffin, and serial sections of 8 μm in thickness were prepared and stained with hematoxylin and eosin. Then, primordial (Pri), type 3 (T3), type 4 (T4), type 5 (T5), type 6 (T6) and type 7 (T7) follicles, were counted. **P < 0.001.

To investigate the effect of *Lkb1* deletion on female fertility, a breeding assay was carried out. As shown in Figure 1C, female *Lkb1^fl/fl^;Gdf9-Cre* mice were significantly subfertile, some of which were completely infertile and some could have offspring with very few litters. To find the reasons for the decreased fertility in mutant mice, we firstly observed the morphology of ovaries from *Lkb1^fl/fl^* and *Lkb1^fl/fl^; Gdf9-Cre* mice. We found that from 1 month of age, ovaries in *Lkb1^fl/fl^;Gdf9-Cre* mice became larger compared to those in *Lkb1^fl/fl^* mice and reached a peak at about 2 months of age (Figure 1D). Further histological analysis of ovaries showed no apparent morphological difference in post-natal day (PD) 16 ovary sections of *Lkb1^fl/fl^* and *Lkb1^fl/fl^;Gdf9-Cre* mice, where both genotypes contained plenty of primordial follicles (Figure 2A-2B and 2B', black arrowheads). However, at PD 35 and 7 weeks of age, respectively, excessive primordial follicles were activated and developed to growing follicles with enlarged oocytes in mutant ovaries (Figure 2D and 2F, yellow arrowheads in *D*' and *F*'), whereas most of the follicles in control ovaries were still at the primordial stage (Figure 2C, black arrowheads; Figure 2E). At 2 months of age, a large number of further developed antral follicles existed in mutant ovaries (Figure 2G-2H, yellow arrowheads in *H*'). Consistent with these observations, quantification analysis of ovarian follicles revealed a significant reduction of primordial follicles and increase of type 3 follicles in PD 35 mutant ovaries (Figure 2I). In addition, we found continuous reduction of primordial follicles but a remarkable increase of other types of follicles, especially of type 5, 6 and 7 follicles in 2-month-old mutant ovaries (Figure 2J). Thus, all the above data confirmed that most of the primordial follicles were prematurely activated in *Lkb1^fl/fl^;Gdf9-Cre* mice by 2 months of age.

### Adult female *Lkb1^fl/fl^; Gdf9-Cre* mice demonstrate follicle depletion and POF

**Figure 3:**
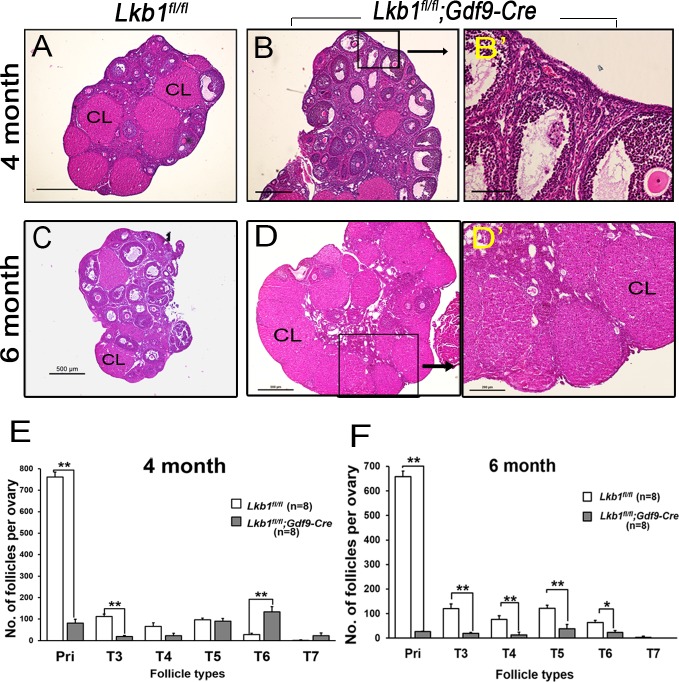
Follicle depletion in adult female *Lkb1^fl/fl^;Gdf9-Cre* mice **A**.-**D**. Histology of ovarian sections from *Lkb1^fl/fl^* and *Lkb1^fl/fl^;Gdf9-Cre* females at 4 months and 6 months of age, respectively, stained with hematoxylin and eosin. Panel B' and D' is a magnification of the rectangular area marked with a solid line in panel **B**. and **D**. CL: corpus luteum. Bars: 200μm in B' and D'; 500μm in **A**.-**D**. **E**.-**F**. Shown are the quantifications of numbers of different types of follicles per ovary at the age of 4 months and 6 months, respectively. Ovaries from *Lkb1^fl/fl^* and *Lkb1^fl/fl^; Gdf9-Cre* mice with indicated ages were embedded in paraffin, and serial sections of 8 μm in thickness were prepared and stained with hematoxylin and eosin. Then, primordial (Pri), type 3 (T3), type 4 (T4), type 5 (T5), type 6 (T6) and type 7 (T7) follicles, were counted. **P < 0.001.

**Figure 4:**
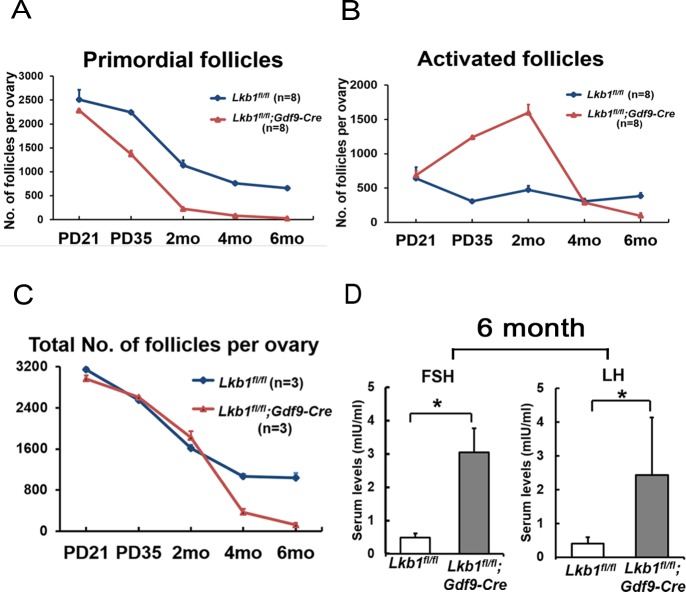
POF in adult female *Lkb1^fl/fl^;Gdf9-Cre* mice **A**.-**B**. Numbers of primordial follicles **A**. and activated follicles **B**. in ovaries of PD21, PD35, 2-month (2 mo), 4-month (4 mo) and 6-month (6 mo)-old *Lkb1^fl/fl^* and *Lkb1^fl/fl^; Gdf9-Cre* mice. Data are shown as mean ± SEM. **C**. Numbers of total follicles per ovary in PD21, PD35, 2-month, 4-month and 6-month (6 mo) old *Lkb1^fl/fl^* and *Lkb1^fl/fl^;Gdf9-Cre* mice. **D**. Shown are the serum levels of FSH and LH. Sera from 6-month-old *Lkb1^fl/fl^;Gdf9-Cre* and *Lkb1^fl/fl^* mice were collected for measurement of indicated hormone levels. Data are shown as mean ± SEM. *P < 0.05.

Compared to *Lkb1^fl/fl^* mice (Figure 3A) at 4 months of age, few primordial follicles or primary follicles were present in the *Lkb1^fl/fl^;Gdf9-Cre* ovaries (Figures 3B and B'). Obviously, the reason for this result was the exhausted primordial follicle pool and continuous growth of activated follicles. In support of this, we found that all follicles including primordial and activated follicles were significantly reduced in *Lkb1^fl/fl^;Gdf9-Cre* mice by 6 months of age (Figure 3C-3D, and D'), which is termed premature ovarian failure (POF). In addition, the 6-month-old mutant mouse ovaries still appeared larger than the control ovaries because of increases in corpora lutea (CL). Quantitative analysis of ovarian follicles revealed less activated follicles of type 3 and type 4 in ovaries of *Lkb1^fl/fl^;Gdf9-Cre* mice but significantly more type 6 and type 7 follicles and a similar number of type 5 follicles compared with *Lkb1^fl/fl^* ovaries at 4 months of age (Figure 3E). However, at 6 months of age, all types of follicles in ovaries of *Lkb1^fl/fl^;Gdf9-Cre* mice were significantly reduced (Figure 3F). In general, the primordial follicle reduction occurred at the onset of sexual maturity (PD35) and subsequently led to follicle depletion during adulthood (6 months) in *Lkb1^fl/fl^;Gdf9-Cre* mice (Figure 4A-4C). In humans, patients with POF are biochemically characterized by high levels of gonadotropins: follicle-stimulating hormone (FSH) and luteinizing hormone (LH) [14]. Here, in sera of 6-month-old mutant mice, elevated levels of FSH and LH (Figure 4D) were observed relative to control mice, suggesting *Lkb1^fl/fl^; Gdf9-Cre* mice as one of the mouse POF models.

### Depletion of LKB1 enhances mTORC1/S6K/rpS6 signaling through AMPK in oocytes

**Figure 5:**
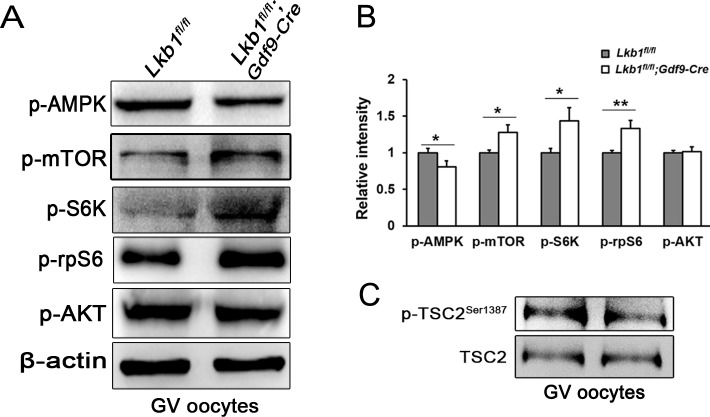
Upregulation of mTORC1 signaling in oocytes of *Lkb1^fl/fl^;Gdf9-Cre* mice **A**. Western blot analyses of p-AMPK (Thr172), p-mTOR (Ser2448), p-S6K (Thr387), p-rpS6 (Ser240/244), p-AKT (Ser473) in GV oocytes from *Lkb1^fl/fl^* and *Lkb1^fl/fl^;Gdf9-Cre* mice. 200 oocytes were used for each lane. β-actin was used as a loading control. All experiments were repeated at least three times and representative images are shown. **B**. Relative intensity of p-AMPK (Thr172), p-mTOR (Ser2448), p-S6K (Thr387), p-rpS6 (Ser240/244), p-AKT (Ser473) with ovary protein extract and GV oocytes from *Lkb1^fl/fl^* and *Lkb1^fl/fl^; Gdf9-Cre* mice. Data are shown as mean ± SEM. *P < 0.05, **P < 0.001. **C**. Western blot analyses of p-TSC2 (Ser1387) and total TSC2 in GV oocytes from *Lkb1^fl/fl^* and *Lkb1^fl/fl^;Gdf9-Cre* mice.

To investigate the mechanisms underlying LKB1-deletion-caused follicular over-activation, we first focused on the mTOR pathway, which balances cell growth with energy control and is also negatively regulated by LKB1 through AMPK. We collected GV oocytes in later stages of follicles from *Lkb1^fl/fl^* and *Lkb1^fl/fl^;Gdf9-Cre* mice as samples for western blot. Results showed that the activity of AMPK (Thr172) was significantly reduced, whereas the activity of the mTORC1/S6K/rpS6 signaling pathway was enhanced, as indicated by the elevated levels of phosphorylated mTOR (Ser2448), phosphorylated S6K (Thr387) and phosphorylated rpS6 (Ser240/244) in the *Lkb1^fl/fl^;Gdf9-Cre* group at 2 months of age (Figure 5A, 5B). In contrast, AKT, an important upstream protein regulating mTOR in the PTEN/AKT/mTOR signaling pathway, which had been proven to govern the activation of primordial follicle pool [5], did not display increased phosphorylation (Ser473) whether in mutant total follicles or GV oocytes (Figure 5A). Additionally, phosphorylated TSC2 (Ser1387), the target of LKB1 and negative upstream regulator of mTORC1, was also decreased (Figure 5C), indicating that the level of mTORC1 activity was elevated by depletion of LKB1 through the LKB1/AMPK signaling pathway.

**Figure 6:**
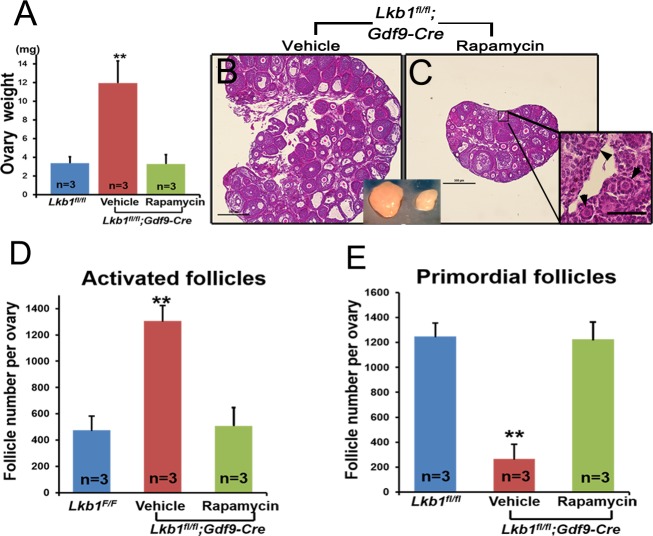
Rapamycin effectively rescues the overactivation of primordial follicles in ovaries of *Lkb1^CKO^* mice **A**. Ovary weights of *Lkb1^fl/fl^* and *Lkb1^fl/fl^;Gdf9-Cre* mice treated with rapamycin or vehicle. **P < 0.001. **B**.-**C**. Histology of ovaries from *Lkb1^fl/fl^;Gdf9-Cre* mice treated with vehicle or rapamycin. Black arrowheads in panel C indicate primordial follicles. Bars: 500μm in panels **B**. and **C**. **D**.-**E**. Numbers of activated follicles **D**. and primordial follicles **E**. in ovaries from *Lkb1^fl/fl^* mice, vehicle-treated *Lkb1^fl/fl^; Gdf9-Cre* mice and rapamycin-treated *Lkb1^fl/fl^; Gdf9-Cre* mice. Data are shown as mean ± SEM. **P < 0.001.

As is known, the mTORC1/S6K/rpS6 signaling pathway is sensitive to the mTORC1 and mTORC2 inhibitor rapamycin [15]. To confirm that it is the up-regulated mTORC1 signaling pathway that leads to the overactivation of primordial follicles in *Lkb1^fl/fl^;Gdf9-Cre* mice, we respectively injected rapamycin and vehicle into *Lkb1^fl/fl^;Gdf9-Cre* mice (3 mg/kg body weight per day) for 1 month, starting at 4 weeks of age. Notably, *Lkb1^fl/fl^;Gdf9-Cre* mice treated with rapamycin showed lower ovary weight when compared to non-treated mutant mice (Figure 6A). Additionally, ovarian morphology analysis showed that in 2-month-old *Lkb1^fl/fl^;Gdf9-Cre* mice treated with rapamycin, typical primordial follicles were found (Figure 6C, arrowheads). On the contrary, in ovaries of mutant mice that were treated with vehicle, most of the primordial follicles had been activated (Figure 6B). Consistent with this phenotype, quantification of follicle numbers showed that the number of primordial follicles in rapamycin-treated *Lkb1^fl/fl^;Gdf9-Cre* mouse ovaries significantly increased, and the number of activated follicles remarkably decreased to a normal level (Figure 6D-6E). These results demonstrate that up-regulated mTORC1 activity by loss of *Lkb1* in oocytes is the major driving force that prematurely activates the primordial follicles in *Lkb1^fl/fl^;Gdf9-Cre* mice, further indicating that proper inhibition of mTORC1 activity in oocytes is necessary for preservation of the primordial follicle pool.

### Oocyte-specific deletion of *Lkb1* from the primordial follicle stage leads to severe female fertility defects due to defective follicular development

**Figure 7:**
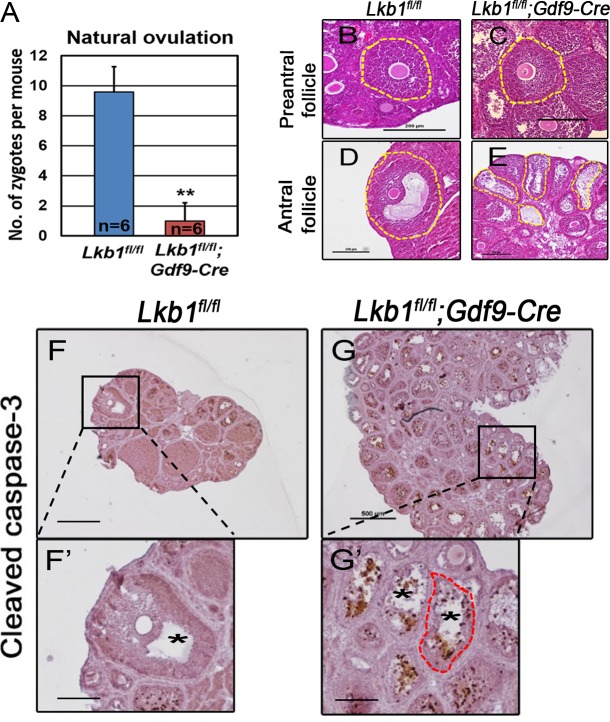
Deletion of *Lkb1* leads to impaired ovulation in *Lkb1^fl/fl^;Gdf9-Cre* mice **A**. Natural ovulation assay shows decreased zygotes collected from *Lkb1^fl/fl^;Gdf9-Cre* female mice. **B**.-**E**. Preantral and antral follicle morphologies in 2-month-old *Lkb1^fl/fl^* and *Lkb1^fl/fl^;Gdf9-Cre* mouse ovaries. Bars: 200μm. **F**.-**G**. Apoptosis analysis of ovaries from 2-month-old *Lkb1^fl/fl^* and *Lkb1^fl/fl^;Gdf9-Cre* mice. Bars: 200μm in F' and **G**.'; 500μm in **F**.-**G**.

**Figure 8:**
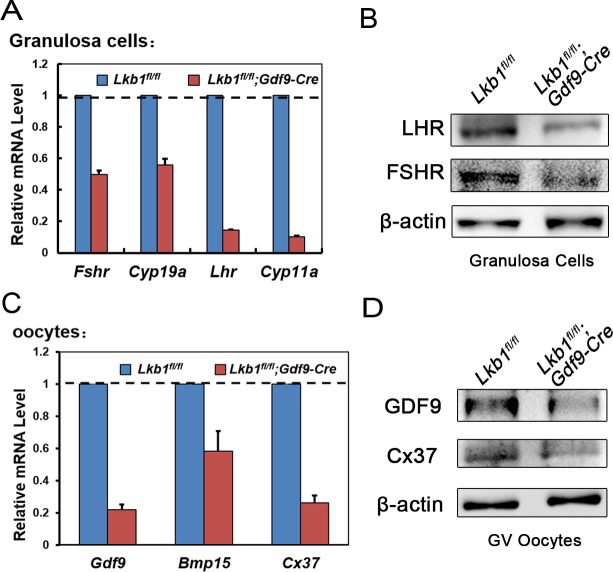
**A.**-**C.** qRT-PCR shows the expression of selected follicular development-related genes in GCs (A) and oocytes (C) in *Lkb1^fl/fl^* and *Lkb1^fl/fl^;Gdf9-Cre* mice. Data are shown as mean ± SEM. **B**.-**D**. Western blot analysis of corresponding protein levels in GCs (B) and oocytes (D) from *Lkb1^fl/fl^* and *Lkb1^fl/fl^;Gdf9-Cre* mice. A total of 200 oocytes were used for each lane.

**Figure 9:**
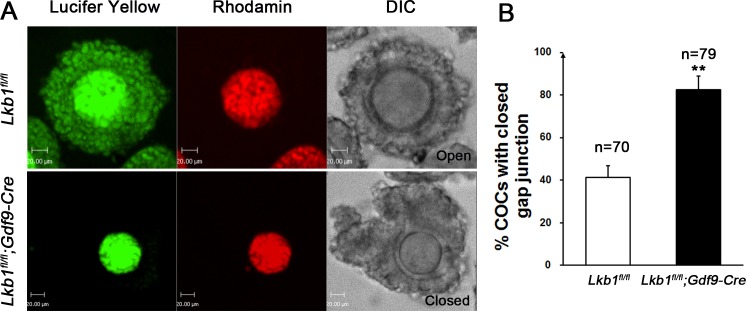
**A.** Dye transfer in the cumulus-oocyte complex. COCs were isolated from *Lkb1^fl/fl^* and *Lkb1^fl/fl^;Gdf9-Cre* mice and oocytes were injected with Lucifer yellow and Rhodamine. Rhodamine, a high molecular weight marker that is not transferred *via* gap junctions, served as a negative control. Bars: 20μm. **B**. Statistical analysis of the COCs with closed gap junction in the indicated mice. Data are shown as mean ± SEM. **P < 0.001.

**Figure 10:**
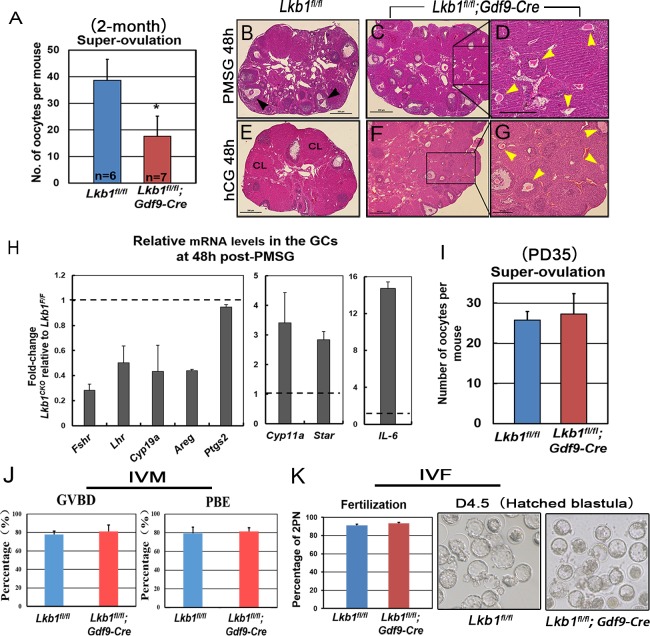
Fewer oocytes superovulated from mutant mice **A**. Superovulation assays show a decrease of oocytes ovulated from 2-month-old *Lkb1^fl/fl^; Gdf9-Cre* female mice. Oocytes were collected from the oviducts of *Lkb1^fl/fl^* and *Lkb1^fl/fl^; Gdf9-Cre* female mice after injection of PMSG (48h) and hCG (16h). *P < 0.05. **B**.-**G**. Hematoxylin and eosin staining of ovaries from 2-month-old *Lkb1^fl/fl^* and *Lkb1^fl/fl^; Gdf9-Cre* female mice treated with PMSG 48h **B**.-**D**. and PMSG 48h & hCG 48h **B**.-**G**. In panel B, black arrowheads indicate preovulatory follicles. In panel **D**. and **G**., yellow arrowheads indicate atretic follicles containing apoptotic oocytes. Bars: 500μm in **B**.-**C**. and **E**.-**F**.; 200μm in **D**. and **G**. **H**. qRT-PCR shows the expression of selected FSH/PMSG and LH/hCG target genes in GCs of 2-month-old *Lkb1^fl/fl^* and *Lkb1^fl/fl^; Gdf9-Cre* mice after PMSG treatment for 48h. Data are shown as mean ± SEM. **I**. Superovulation assays show normal ovulation rate of *Lkb1^fl/fl^; Gdf9-Cre* female mice at PD35. Oocytes were collected from the oviducts of *Lkb1^fl/fl^* and *Lkb1^fl/fl^; Gdf9-Cre* female mice after injection of PMSG (48h) and hCG (16h). **J**. *In vitro* maturation (IVM) of GV oocytes from *Lkb1^fl/fl^* and *Lkb1^fl/fl^;Gdf9-Cre* mice ovaries. Total numbers of oocytes undergoing germinal vesicle breakdown (GVBD) and polar body extrusion (PBE) were counted under the microscope at 2h and 12h, respectively. **K**. *In vitro* fertilization (IVF) of MII oocytes super-ovulated from *Lkb1^fl/fl^* and *Lkb1^fl/fl^;Gdf9-Cre* mouse ovaries. After fertilization, the zygotes were cultured for further development.

With so many growing follicles existing in the ovaries, the mutant mice should have more offspring than the control mice. But on the contrary, they were almost sterile, why? To confirm whether such subfertility was due to an ovulation problem, we next compared the numbers of naturally ovulated eggs in control and mutant mice by counting zygotes collected from oviducts after successful mating. Surprisingly, although excessive follicles developed, the average number of naturally ovulated eggs from *Lkb1^fl/fl^;Gdf9-Cre* mice was significantly less than that of *Lkb1^fl/fl^* mice (Figure 7A), which coincided with the results from the breeding assay. Further histological analysis showed that the large antral follicles were severely impaired in the ovaries of 2-month-old mutant mice, while the preantral follicles showed normal morphology (Figure 7B-7E). Correspondingly, the expression of selected marker genes related to follicular development in granulosa cells (GCs) (*Fshr*, *Lhr*, *Cyp19a* and *Cyp11a)* and oocyte-derived factors that affect follicular development including gap junctions (*Gdf9, Bmp15 and Cx37*) [16] from *Lkb1^fl/fl^;Gdf9-Cre* mice were all dramatically decreased (Figure 8A-8D), which indicated defects in follicular maturation. Additionally, to confirm the defective communication between oocytes and cumulus cells (CCs) in large antral follicles of mutant mice, we employed dye transfer experiments and found that gap junctions in 82% of Lucifer yellow-injected oocyte-cumulus cell complex (COC) from mutant mice were closed, in contrast to 41% in the control group (Figure 9A-9B). More surprisingly, when we employed superovulation to induce ovulation artificially with exogenous PMSG and hCG, the mutant mice still ovulated less than the control mice (Figure 10A) because most antral follicles in mutant mice initiated atresia by premature luteinization (Figure 10C-10D) and formed numerous atretic corpora lutea (CLs) with degenerated oocytes inside (Figure 10F-10G) instead of developing into preovulatory follicles (Figure 10B) and forming healthy CLs after ovulation (Figure 10E), which was also confirmed by the abnormal expression of relevant genes in GCs (Figure 10H). All these data suggest that most of the antral follicles of *Lkb1^fl/fl^;Gdf9-Cre* mice had defects and would undergo atresia, instead of being selected as dominant follicles and maturing to the preovulatory stage, which could explain why the mutant mice ovulated fewer oocytes.

**Figure 11:**
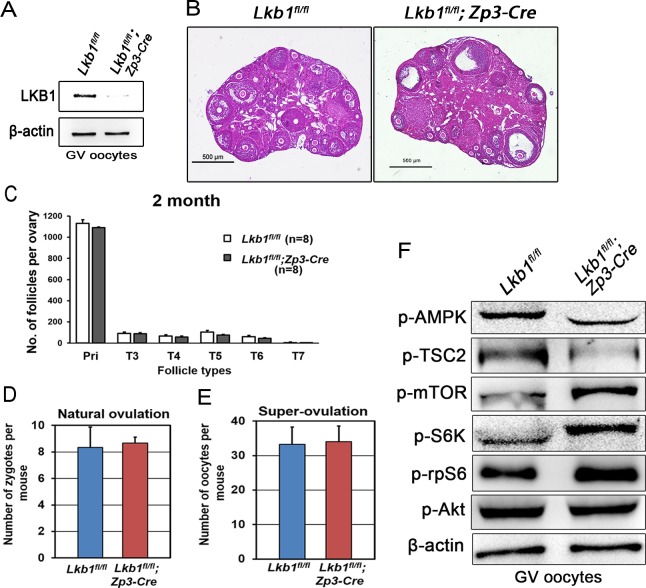
Follicular development is not affected in *Lkb1^fl/fl^;Zp3-Cre* mice **A**. Western blot showing the depletion of LKB1 from oocytes in *Lkb1^fl/fl^;Zp3-Cre* mice. For each lane, 200 oocytes were used. **B**. Histology of ovaries from 2-month-old *Lkb1^fl/fl^* and *Lkb1^fl/fl^;Zp3-Cre* female mice. Bars: 500μm. **C**. Shown are the quantifications of numbers of different types of follicles in 2-month-old *Lkb1^fl/fl^* and *Lkb1^fl/fl^; Zp3-Cre* mouse ovaries. **D**. Natural ovulation assay shows no significant difference in zygote numbers between *Lkb1^fl/fl^* and *Lkb1^fl/fl^;Zp3-Cre* female mice. **E**. Superovulation assay shows no obvious difference in oocyte numbers between 2-month-old *Lkb1^fl/fl^* and *Lkb1^fl/fl^; Zp3-Cre* female mice. Oocytes were collected from the oviducts of *Lkb1^fl/fl^* and *Lkb1^fl/fl^; Zp3-Cre* female mice after injection of PMSG (48h) and hCG (16h). **F**. Western blot analyses of p-AMPK (Thr172), p-TSC2 (Ser1387) p-mTOR (Ser2448), p-S6K (Thr387), p-rpS6 (Ser240/244), p-AKT (Ser473) in GV oocytes from *Lkb1^fl/fl^* and *Lkb1^fl/fl^;Zp3-Cre* mice.

**Figure 12:**
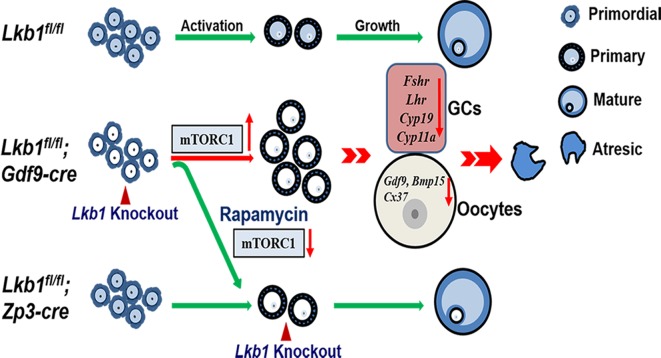
Schematic diagram of follicular development in *Lkb1^fl/fl^, Lkb1^fl/fl^;Gdf9-Cre* and *Lkb1^fl/fl^;Zp3-Cre* mouse ovaries In normal mice (*Lkb1^fl/fl^*) ovaries, only a limited number of primordial follicles are recruited into the growing follicle pool. When we deleted Lkb1 from primordial follicle oocytes (*Lkb1^fl/fl^;Gdf9-Cre*), excessive primordial follicles were activated with elevated mTORC signaling pathway, resulting in defective follicular development. After rapamycin treatment, overactivation of primordial follicles in *Lkb1^fl/fl^;Gdf9-Cre* mice could be rescued to be the normal level. Additionally, when we deleted LKB1 from oocytes in the primary follicle stage (*Lkb1^fl/fl^;Zp3-Cre*) to avoid overactivation, no defects were observed in the following follicular develop.

The sharp contrast between the large number of growing follicles existing in the ovaries of mutant mice and the few oocytes ovulated still was surprising and provoked an obvious question: which mechanism(s) caused the defective follicular selection? Based on the following facts about the mutant mice: (1) unlike large antral follicles, the follicles at preantral and early antral stages still showed normal morphology (Figure 7C, 7E); (2) the morphological anomalies of later follicular development could be rescued by rapamycin which inhibited the follicular over-activation (Figure 6C); (3) in the superovulation test, exogenous gonadotropins could help solve the ovulation problem of 2-month-old mutant mice to some extent (Figure 10A), moreover, at PD35 when the phenotype just begins to show, the mutant mice still could ovulate normally like the control (Figure 10I); therefore, we came up with a bold speculation that it was not the *Lkb1* deletion but the competition effects of too many follicles growing at the same time that impaired the follicular selection directly. To further prove this, we first collected GV oocytes from developed follicles of 2-month-old *Lkb1^fl/fl^; Gdf9-Cre* mice to perform *in vitro* studies. As we expected, most of the fully grown mutant GV oocytes could mature normally with average GVBD and PBE rates (Figure 10J). Besides, the mature eggs superovulated from mutant mice could be fertilized by IVF and developed to hatched blastula stages with no significant difference to the control groups (Figure 10K). To be more convincing, we then employed *Zp3-Cre* to specifically delete *Lkb1* in oocytes from the primary follicle stage on to exclude the side-effects from over-activation of primordial follicles (Figure 11A). Histological analysis of ovaries showed no apparent morphological difference between *Lkb1^fl/fl^* and *Lkb1^fl/fl^; Zp3-Cre* mice (Figure 11B), in agreement with the follicle counting result (Figure 11C). Moreover, although the AMPK/TSC2/mTORC1/S6K/rpS6 signaling pathway was also enhanced in the GV oocytes from the *Zp3-cre* mutant mice (Figure 11F), *Lkb1^fl/fl^; Zp3-Cre* mice ovulated no fewer oocytes than *Lkb1^fl/fl^* mice in both natural ovulation and superovulation experiments (Figure 11D-11E), suggesting that LKB1 depletion in oocytes from the primary follicle stage does not affect follicular growth, selection or ovulation at all. All the above evidence points to the conclusion that the main *in vivo* function of LKB1 in oocytes is to serve as gatekeeper of the primordial follicle pool rather than regulating ovulation (Figure 12), and there is a stage-specific function of LKB1-AMPK signaling in mouse oocytes that controls follicular activation.

## DISCUSSION

The molecular mechanisms that govern quiescence and activation of primordial follicles remained poorly understood for decades until genetically modified mouse models recently provided some clues. Earlier reports have shown that Foxo3a, p27, PTEN, TSC/mTORC1 signaling are important brakes that restrain follicular activation [5, 17-21]. In the current study, by using a mutant mouse model with oocyte-specific deletion of *Lkb1*, we show that LKB1 in oocytes is indispensable for the preservation of dormant primordial follicles by suppressing follicular activation.

Interestingly, our *Lkb1* mutant mouse model and *Pten* or *Tsc1/2* mutant mouse models share many common features. First, the deletion of these genes in oocytes leads to premature activation of the entire primordial follicle pool resulting in POF. Second, besides the phenotypic similarities, enhanced mTORC1-S6K-rpS6 signaling is responsible for the over-activation of primordial follicles in all the three mutant mouse models. However, oocyte-specific knockout of *Lkb1* is different from that of *Pten* or *Tsc1/2* in several ways. On one hand, LKB1 in oocytes controls follicular dormancy and activation in a distinct way compared to PTEN. The loss of LKB1 in oocytes led to enhanced mTORC1-S6K-rpS6 signaling without change in AKT activity but with reduced AMPK activity (Figure 5), which suggests that LKB1 targets downstream mTORC1 through AMPK [7], unlike PTEN through AKT [5]. Thus, LKB1 may mediate a new signaling pathway to control the follicular activation. On the other hand, unlike the phenotypes in oocyte-specific deletion of *Pten*, *Tsc1* or *Tsc2* ovaries where most of overactivated follicles show quick demise [5, 17, 18], almost all of the activated follicles in *Lkb1^fl/fl^;Gdf9-Cre* ovaries survive to the antral follicle stage (Figure 2), which might be due to the normal level of AKT activity in PTEN/PI3K signaling which also determines survival of primordial follicles [5]. Thus, like PTEN, LKB1 regulates primordial follicle dormancy/activation; unlike PTEN, LKB1 does not seem to be responsible for oocyte growth, indicating that these two pathways may play different roles in growing follicle development. As the direct upstream regulator of mTORC, TSC1/2** could function more efficiently in the regulation of mTORC1 pathway than *Lkb1*. Though the deletion of LKB1 resulted in an up-regulated mTORC pathway by reduced activities of AMPK and TSC2, it might still not be sufficient to affect the survival of these activated follicles. Moreover, the PI3K-AKT pathway regulated by PTEN, is actually a multifunctional pathway which has many other downstream targets besides to mTORC1, thus it is likely that PTEN regulates oocyte survival and growth through other targets of AKT more than just mTORC1. Therefore, it is reasonable to conclude that the LKB1-AMPK axis is a weaker regulator of mTORC1 activation compared to the PTEN-AKT and TSC1/2 axis in the survival and development of ovarian follicles. Moreover, deletion of *Lkb1* in oocytes does not have an immediate effect on follicular activation until after PD21 around the time for onset of sexual maturity, which suggests that the LKB1-AMPK pathway might be another driving force that restrains follicular activation after puberty, cooperating with the PTEN/PI3K-AKT pathway. There is also a possibility that it may take some time for the degradation of LKB1 protein and downregulation of LKB1-mediated signals or that some temporary compensatory mechanisms might take over the functions of LKB1 signaling before sexual maturity.

In mammalian cyclic follicular recruitment, only one or a limited number of growing follicles are selected as the dominant follicles which can escape from atresia [22] to ultimately develop to preovulatory follicles and determine the number of ovulated eggs. It has been reported that all the recruited follicles during cyclic recruitment are characterized by expression of mRNAs encoding a range of steroidogenic enzymes, gonadotrophin receptors and local regulatory factors such as IGFs and the TGFβ superfamily (inhibins, activins and bone morphogenetic proteins (BMPs)) and the well differentiated follicles will be selected by the rise and fall of FSH levels [23, 24]. During this process, bi-directional communication between oocytes and the companion granulosa cells is essential [16, 25]. In our study, most of the activated follicles in mutant mouse ovaries could survive and grow (Figure 2 and Figure 4), but few of them were finally able to ovulate eggs with defective expression of marker genes both in oocytes and GCs (Figure 7). This suggests that few activated follicles could be selected as dominant follicles to develop to mature preovulatory follicles. As for why LKB1 depletion in *Lkb1^fl/fl^;Gdf9-Cre* oocytes impairs later follicular development, we reasoned that this might be due to a competitive growth among antral follicles in the mutant mouse ovary - the side effect of follicular overactivation. Since so many activated follicles are growing in one ovary at the same time, there might be competition effects among the follicles for the limited resource, causing all being harmed as the outcome. Given that the differentiation of GCs is initiated and maintained by gonadotrophins, it is reasonable to suppose that the deficient differentiation of GCs was the result of the relative deficiency of gonadotrophins for every single follicle. So we treated the mutant mice with exogenous gonadotropins and it did help to solve the ovulation problem to some extent. We also proved this hypothesis by depletion of LKB1 in oocytes from the primary follicle stage which did not lead to over-activation of primordial follicles and follicular defects (Figure 9C-9E) which, together with the results of rapamycin rescue experiment and the normal ovulation rate of PD35 mutant mice, suggested that without follicular over-activation, the absence of LKB1 in oocytes does not affect the later follicular development. Besides, the fact that fully grown GV oocytes collected from *Lkb1^fl/fl^;Gdf9-Cre* ovaries could complete meiotic maturation, be fertilized and develop normally to the hatched blastula stage *in vitro* (Figure 9A-9B) indicates that the follicular defects in the mutant mice were not due to the developmental competence of oocytes. Taken together, all lines of evidence prove the physiological role of intra-oocyte LKB1 to be a critical gatekeeper of the ovarian primordial follicle pool.

For decades, the inhibitory system for the resting primordial follicles has remained unknown. But over the past 6 years, much progress has been made by using oocyte-specific knockout mouse models. Furthermore, these findings from mouse models have been successfully translated to clinical treatment of patients. As the PTEN inhibitor and AKT stimulator have been used for *in vitro* activation of primordial follicles [26] and even healthy babies were born already [27], we may also consider the application of an AMPK inhibitor, for example, Compound C, in the *in vitro* activation of primordial follicles. This application might be more efficient since the down-regulation of LKB1-AMPK pathway would not affect the survival of activated follicles and mature eggs could be obtained in an easier way.

In conclusion, intra-oocyte LKB1 is a critical regulator of primordial follicle dormancy/activation. Recognition of the role of the LKB1-AMPK signaling network in oocytes may open up new prospects for understanding of the physiological and pathological processes of the mammalian ovary. Our work may provide valuable information for the design of therapeutics for POF.

## MATERIALS AND METHODS

### Mice

*Lkb1^fl/fl^;Gdf9-Cre* and *Lkb1^fl/fl^;ZP3-Cre* mice were respectively generated by crossing *Lkb1^fl/fl^* mice (FVB/n background) with *Gdf9-Cre* mice and *ZP3-Cre* mice (C57BL/6J background). Animals were backcrossed at least for four generations onto an FVB/n background for the experiments. The mice were housed under controlled environmental conditions with free access to water and food. Animal care and handling were conducted according to the guidelines of the Animal Research Committee of the Institute of Zoology, Chinese Academy of Sciences.

### Antibodies

Antibodies used in the experiments were purchased from the following companies: Rabbit monoclonal anti-phospho-AMPK (Thr172), anti-phospho-mTOR (Ser2448), anti-phospho-riboprotein S6 (Ser240/244), anti-phospho-p70S6K (Thr387), anti-phospho-akt (Ser473), anti-phospho-TSC2 (Ser1387), anti-TSC2, anti-cleaved caspase 3 (CST; Beverly, MA, USA); Goat polyclonal anti-LKB1, Rabbit polyclonal anti-FSHR, LHR, Cx37, Gdf9 (Santa Cruz; Dallas, TX, USA). Mouse monoclonal anti-β-actin and secondary antibodies were purchased from ZhongShan Golden Bridge Biotechnology Co., LTD (Beijing, China).

### Histological analysis and immunohistochemistry

Ovaries used for histological analysis were fixed in 4% paraformaldehyde (pH 7.5) overnight at 4°C, dehydrated, and embedded in paraffin. Paraffin-embedded ovaries were sectioned at a thickness of 8-μm for hematoxylin and eosin staining. Immunohistochemistry was performed on 8-μm sections using the Vectastain ABC kit (Vector Laboratories, CA, USA). For direct comparison, ovary sections from four individual females of each genotype were processed together.

### Quantification of ovarian follicles

Quantification of ovarian follicles was performed as previously described by Kui Liu in 2007 [28]. Briefly, to count the numbers of follicles, paraffin-embedded ovaries were serially sectioned at 8-μm thickness and every fifth section was mounted on slides. Then these sections were stained with hematoxylin and eosin for morphological analysis. Ovarian follicles at different developmental stages, including primordial, primary (type 3 and type 4), secondary (type 5), and antral follicles (type 6 and type 7) were counted in collected sections of an ovary, based on the well-accepted standards established by Peterson and Peters [29]. In each section, only those follicles in which the nucleus of the oocyte was clearly visible were scored and the cumulative follicle counts were multiplied by a correction factor of 5 to represent the estimated number of total follicles in an ovary.

### Western blot analysis

Ovary lysate was prepared from minced ovaries after removal of suspended granulosa cells by centrifugation for western blot analysis. 30μg ovary protein or 200 oocytes were mixed with SDS sample buffer and boiled for 5 min at 100°C for SDS-PAGE. Western blot was performed using antibody dilutions recommended by the manufacturer. HRP-labeled secondary antibody was used at a dilution of 1:3000.

### RNA isolation and quantitative real-time PCR analysis

Total RNA from GCs was extracted using RNeasy micro purification kit (Qiagen; Hilden, Germany). Single-stranded cDNAs were generated with cDNA synthesis kit (Invitrogen; Carlsbad, CA, USA). Real-time PCR was conducted by using SYBR Premix Ex Taq^TM^ kit (TaKaRa Biotechnology (Dalian) Co., Ltd., Japan) in ABI prism 7500 Sequence Detection System. Analysis of relative gene expression was measured by quantitative real-time PCR and the 2^−ΔΔCT^Method.

### *In vivo* treatment of mice with rapamycin

For daily intraperitoneal injection of the mice, a dosage of 3 mg/kg body weight was used. The *Lkb1^fl/fl^;Gdf9-Cre* mice were injected daily from 4-weeks to 8-weeks of age and then killed. Mice injected with vehicle were used as controls.

### Breeding assays

In breeding assays, *Lkb1^fl/fl^* and *Lkb1^fl/fl^;Gdf9-Cre* genotype female mice with sexual maturity were continually mated to *Lkb1^fl/fl^* male mice with known fertility for 6 months. Cages were checked daily for counting the number of litters and pups.

### Natural ovulation and gonadotrophin-induced ovulation

For the natural ovulation assay, 2~4 month-old female mice were examined for the estrous cycle every day and females in the estrus were mated with a reproductive male. The next morning, the oviducts were dissected from the euthanized females, and zygotes were collected, counted and analyzed. To induce synchronized follicular growth and ovulation, each female mouse at the indicated age was injected with 7.5 IU PMSG followed by 7.5 IU hCG after 48h to promote ovulation. Mice were killed at 16h of hCG treatment and cumulus-oocyte complexes were recovered from each oviduct. After a 5-min treatment with 1mg/ml hyaluronidase (Sigma, St. Louis, MO, USA) in M2 medium, oocytes were collected and counted.

### GV oocyte collection, culture, fertilization *in vitro* and granulosa cell isolation

Germinal vesicle (GV)-stage oocytes were isolated from ovaries of 9 week-old female mice and cultured in M2 medium (Sigma, St. Louis, MO, USA) under paraffin oil at 37°C, 5% CO_2_ in air. Mature oocytes were fertilized *in vitro* by a routine procedure, and fertilized eggs were further cultured *in vitro*. For granulosa cell isolation, adult female mice (~9 weeks) were injected with 7.5 IU PMSG, and ovaries were harvested after 48h. Ovaries were dissected and placed in DMEM. Large antral follicles were punctured to extrude granulosa cells, which were then strained and spun down. For each independent experiment, more than three mice with indicated genotypes were used in each group.

### Dye transfer

COCs isolated from ovaries of PMSG-primed female mice were microinjected with 5% Lucifer yellow (Molecular Probes, Eugene, OR) and rhodamine dextran for 1 min. Injections resulted in dye filling the oocyte and further spreading to the cumulus cells through gap junctions. Rhodamine dextran, which cannot pass the gap junction channels due to its high molecular weight, confirmed the spread of dye observed reflected transfer through gap junctions. The experiments were recorded with a Perkin Elmer precisely Ultra VIEW VOX Confocal Imaging System (PerkinElmer, Waltham, MA, USA).

### Serum analysis

Mice were anesthetized for blood collection by eyeball removal. FSH and LH measurements were conducted at a commercial laboratory (Chemclin Co., Beijing, China). At least 8 mice were anesthetized for each group.

### Statistical analysis

All experiments were repeated at least three times. Statistical analysis was performed using SPSS. Data were expressed as mean ± SEM and *p* < 0.05 was considered statistically significant.
